# Coping with Coping: International Migrants’ Experiences of the Covid‐19 Lockdown in the UK

**DOI:** 10.1111/1467-8551.12512

**Published:** 2021-05-25

**Authors:** Dorothy Ai‐wan Yen, Benedetta Cappellini, Hsiao‐Pei (Sophie) Yang, Suraksha Gupta

**Affiliations:** ^1^ Brunel Business School Brunel University London Kingston Lane Uxbridge UB8 3PH UK; ^2^ University of Durham Stockton Road Durham DH1 3LE UK; ^3^ Coventry University Priory Street Coventry CV1 5FB UK; ^4^ Newcastle University London 102 Middlesex Street London E1 7EZ UK

## Abstract

Globally, policymakers have overlooked the challenges faced by international migrants in host countries during the Covid‐19 pandemic. The policies and support systems designed by host governments highlight the lack of social justice and raise concerns for scholarly attention. Considering the experiences of international migrants living in the UK during the Covid‐19 lockdown from the theoretical perspective of coping, this interpretivist study investigates international migrants’ coping strategies adopted during the first UK national lockdown. Data collected from 60 Chinese, Italian and Iranian migrants using semi‐structured interviews during the lockdown period were analysed thematically using NVivo. The findings show that migrants adopted multi‐layered and multi‐phase coping strategies. To cope with the anxiety and uncertainties caused by the pandemic, they initiated new practices informed by both home and host institution logics. Nevertheless, the hostile context's responses provoked unexpected new worries and triggered the adoption of additional and compromising practices. The paper illustrates how coping became paradoxical because migrants had to cope with the hostile reactions that their initial coping strategies provoked in the host environment. By introducing the new concept of *coping with coping*, this paper extends previous theoretical debate and leads to several managerial implications for governments and policymakers.

## Introduction

Covid‐19 as a global pandemic has disrupted the life of everybody. Governments in every country are taking a lead in imposing disease prevention and control measures, employing various interventions to prevent the transmission of Covid‐19 and to minimize its damage on the nation. These include communication campaigns, social distancing measures, national and regional lockdowns, financial support schemes for companies and employees, and particular advice targeting the more vulnerable groups, including the elderly and people at high risk, who have been required to shield. Nevertheless, Covid‐19 does not affect all groups of people equally.

Reports show that migrants tend to suffer and struggle to cope more than non‐migrants during global pandemics, yet policy interventions and explanations by government officials have often overlooked international migrants (Kumar *et al*., 2020; Nanthini, 2020). Table 1 illustrates that international migrants are likely to suffer more from Covid‐19 than non‐migrants because of cultural barriers, reduced access to healthcare and welfare, limited social and often economic capital and working conditions (Grierson, 2020; Kumar *et al*., 2020; Migration Data Portal, 2020; Nanthini, 2020; World Bank, 2020). Understanding the conditions of migrants is a pressing matter that demands more research attention and requires urgent government intervention to increase social justice, integration and harmony.

**Table 1:** Covid‐19's negative impact on international migrants

Covid‐19 and international migrants
International migrants, who are not born in the country, have challenging language barriers and would require more time and energy to process, interpret, understand and make sense of new policies, including disease prevention and control rules and regulations, than non‐migrants.
International migrants often come to a host country alone and do not have family support available to them in the host country. They may struggle more to cope emotionally without immediate family support than non‐migrants.
International migrants tend to have limited access to the full healthcare system and many of them do not have healthcare insurance cover, compared to non‐migrants.
International migrants are likely to suffer from higher stigmatization, and to exacerbate xenophobia and discrimination compared to non‐migrants. This was evidenced in the rising of hate crime against migrants in countries such as the UK and USA.
International migrants are more likely to be exposed to the coronavirus, as they tend to be concentrated in urban economic centres (cities).
International migrants are generally more vulnerable to risks of unemployment during an economic crisis.
International migrants are less resourceful and often do not have access to full financial services and remittance support available, compared to non‐migrants. Poorer and irregular international migrants often do not have access to online digital payment platforms.
International migrants make a significant contribution as a labour force to the healthcare sector. As key workers, such as doctors and nurses, they endure higher risks of disease infection. Migration Data Portal (2020) shows that in the UK, foreign‐born healthcare professionals contribute a significant portion of the healthcare system – 33.1% of doctors were foreign‐born, while 21.9% of nurses were foreign‐born.

*Sources*: Grierson (2020); Kumar *et al*. (2020); Migration Data Portal (2020); Nanthini (2020); World Bank (2020).

Overlooking international migrants in government policy responses is short‐sighted, considering their roles in the cultural and social life of countries as well as their contributions as a (or ‘to the’) workforce (World Bank, 2020). While some highlight how international migrants might be less familiar with the host country's institutional logics and may not have fully developed a cognitive map to make sense of both formal and informal rules of action and governmental measures (Scott *et al*., 2000; Thornton and Ocasio, 2008), others point out that they might also experience barriers, including racism and xenophobia, preventing them from accessing health care services and other forms of support (Crockett and Grier, 2021). Achieving effective disease prevention and control without considering different migrant groups’ experiences, understanding and reactions to the imposed measures and interventions may create possible gaps in the epidemic prevention for government management (World Bank, 2020).

Acknowledging this pressing matter, this interpretivist study asks the research question: *how do international migrants living in the UK cope with Covid‐19 and the lockdown measures imposed by the UK government?* To address this research question and help bring social justice to international migrants, we focus on exploring their experiences of living in the UK during the Covid‐19 lockdown, by analysing their coping strategies through the adoption of various practices during the mandatory confinement, and their understanding of the UK government's intervention measures. We refer to the interdisciplinary literature on coping strategies (Jin, 2009, 2010; Kolier, 1991; Lazarus and Folkman, 1984; Somech and Drach‐Zahavy, 2007), paying particular attention to studies offering a less individual‐driven approach to coping with crises and investigating the social and cultural contexts wherein individuals are embedded (Moroşanu and Fox, ; Rzepnikowska, 2019). Understanding coping strategies in specific contexts (international migrants in the UK) is particularly timely, since the UK has suffered a high death toll and the government's Covid‐19 measures and strategies have received controversial global reviews and scrutiny (Henley, 2020). Specifically, we chose to explore three different migrant groups and their coping experiences during the lockdown. Migrants from China, Italy and Iran were selected, as these three countries had suffered the highest number of deaths at the time when the data collection took place.

In total, 60 semi‐structured interviews were conducted with Chinese, Italian and Iranian migrants in the UK. Findings reveal multi‐layered integrated coping strategies, including individual, household‐based and community‐based strategies, adopted by the migrants to cope with the anxieties and uncertainties caused by the pandemic. Our findings also show that international migrants’ coping strategies are context‐specific, informed by both home and host institutional logics. As such, there are some attitudinal and behavioural differences amongst the Chinese, Italian and Iranian sample groups. Some coping strategies, learnt from their home (country), triggered negative responses from the hostile context, which provoked new anxieties in participants, leading to their deployment of additional and/or ad‐hoc solutions that are deemed unsatisfactory and compromising, exacerbating migrants’ marginalization.

Theoretically, this paper advances the understanding of coping by unfolding a new paradoxical coping concept, which we refer to as *coping with coping*, and its idiosyncrasies. It illustrates how migrants navigate through the often‐conflicting home versus host government institutional logics and why they had to compromise their coping strategies in response to the hostile reactions within the host country. As such, we argue that rather than reducing anxiety and uncertainties, coping with coping often leads to further emotional strains, stress and cognitive dissonance, affecting migrants’ wellbeing. Our research findings have implications for government policymakers to help reduce the paradoxical coping difficulties that migrants experience. Action should be taken to better understand individual migrants’ coping strategies, to support migrant families and communities, and to promote societal understanding and inclusion of various practices, informed by different institutional logics.

## Literature review

### An interdisciplinary perspective on coping

The concept of coping, which originated in clinical psychology, has been defined as ‘constantly changing cognitive and behavioural efforts to manage specific external and/or internal demands that are appraised as taxing or exceeding the resources of the person’ (Lazarus and Folkman, 1984, p. 141). A review of interdisciplinary literature reveals that coping with a stressful situation is conceptualized around the assumptions aimed at managing rather than controlling such a situation. Thus, coping implies individuals evaluating the situation and mobilizing behavioural and cognitive efforts to interact within their environment (Lazarus and Folkman, 1984; Rückholdt *et al*., 2019; Somech and Drach‐Zahavy, 2007). Following these assumptions, coping responses have been identified as problem‐focused strategies (directed at remedying external obstacles), emotion‐focused strategies (managing the emotional responses to such obstacles) and avoidance‐focused strategies (including attempts to remove oneself from such obstacles) (Duhachek, 2005; Kashdan *et al*., 2006). Studies also conclude that all coping strategies involve stressful experiences and that while problem‐focused strategies and emotion‐focused strategies are related to a positive adaptation to a stressful situation, avoidance‐focused strategies can be related to both positive and negative adaptation (Duhachek and Kelting, 2009; Han, Duhachek and Rucker, 2015).

While these studies are crucial in understanding individual responses to crises, conflicts and obstacles, they have been criticized for looking at individualized strategies, downplaying the social and cultural context where individuals are embedded (Afifi, Hutchinson and Krouse, 2006; Bruce and Banister, 2019). Recent studies show the importance of the crisis that individuals seek to manage. Jin (2009, 2010) argues that emotional responses to crises vary depending on the individual understanding of the situation. While anxiety is common in all types of crises, people will experience anger in controllable and predictable crises, sadness in predictable and uncontrollable crises, and fright in unpredictable and out‐of‐control crises (Jin, 2010). Studies looking at how migrants cope with stressful situations, including experiences of racism, argue that emotion‐focused strategies are interlinked with cognitive appraisals of their own identities (Berjot and Gillet, 2011). Commonly used strategies include individuals’ selective affiliation to their ethnic group and decreasing their identification with their ethnic communities (Berjot and Gillet, 2011; Moroşanu and Fox, ; Rzepnikowska, 2019).

Some studies point out the relational nature of coping strategies (Hallier, 2000; Korczynski, 2003; Vlaisavljevic, Cabello‐Medina and Pérez‐Luño, 2016) that individuals enact to protect themselves and significant others from financial hardship (Cappellini, Marilli and Parsons, 2014; Hutton, 2016), illness (Kolier, 1991) and racism and discrimination (Moroşanu and Fox, ; Rzepnikowska, 2019). In particular, Hutton (2016) shows how relational coping strategies are crucial for developing resilience to crises and improving self‐worth and a sense of empowerment. Consumer studies looking at these strategies identify commonly used strategies, including consuming brands which will be appreciated by peers (Hamilton, 2009), changing services and products perceived as inadequate and adopting technological devises supporting easier consumption within the family (Falchetti, Canniatti Ponchio and Poli Botelho, 2016).

Others have also shown the existence of communal coping strategies where community members adopt strategies incorporating both individual and more collective approaches to their daily enactments of coping (Baker and Baker, 2016). Such approaches provide members with practical and emotional support which make their networks a community of coping, in which relational strategies become very effective at individual and collective levels (Bruce and Banister, 2019). This seems to be echoed by studies showing how community support is key in strengthening the coping strategies of migrants, who have been victims of racism (Jiang and Korczynski, 2016; Mak and Nesdale, 2001; Tervonen and Enache, 2017).

Taking inspiration from the last set of works that challenge the usefulness of an individual‐based approach while advocating for a more context‐based understanding of coping, this paper seeks to understand international migrants’ coping strategies as a fluid process in which individual, relational and contextual factors intersect. As a fluid process, coping strategies are affected by the local environment (Bruce and Banister, 2019; Lawrence and Callan, 2011), gender (Hutton, 2016), social class (Cappellini, Marilli and Parsons, 2014) and ethnicity. While studies on coping strategies with regard to class and gender are emerging, less is known about ethnicity or nationality (Mak and Nesdale, 2001), and many have called for further studies to investigate how international migrants overcome difficulties and challenges in a host country and how they might enact coping strategies (Moroşanu and Fox, ; Rzepnikowska, 2019; Tervonen and Enache, 2017). As we know from the literature, individuals modify their consumer behaviour as a coping strategy (Cappellini, Marilli and Parsons, 2014; Hamilton, 2009; Hutton, 2016); thus, having a more in‐depth understanding of how migrants might have adapted their practices during the pandemic is a way of answering this call for further studies.

### Government institutional logic and pandemic management

Institutional logics, defined as ‘the socially constructed, historical patterns of material practices, assumptions, values, beliefs, and rules’ (Thornton and Ocasio, 1999, p. 804), influence how individuals behave, produce and reproduce material subsistence and organize time and space, eliciting meaning to the understanding of the social reality. Institutional logics offer both formal and informal rules of action, interaction and interpretation, providing the ‘cognitive maps’ (Scott *et al*., 2000, p. 20) and guidance to organization behaviours and decisions. Government, as leading national institutions, make laws, rules and regulations, collect taxes and print money, acting on behalf of people's interests, while enforcing rules of appropriate citizen behaviour, which reflect the nation's institutional logics and cognitive mapping.

The national healthcare system is a primary area where societal‐level logics created by government are embodied in policies and procedures that cascade down (Currie and Guah, 2007). Existing literature shows that national government institutions are key players in preventing and controlling pandemics, through policymaking and regulation enforcement. Often, the power is held centrally by national governments, with scientific advice offering unquestionable policy legitimacy (Chambers, Barker and Rouse, ; Hajer, 2003), although more recent work suggests that enhanced cooperation between multiple stakeholders, including the state, local governments, businesses and non‐profit organizations, may provide a more effective and holistic approach to pandemic disease control and management (Schwartz and Yen, 2017).

A government's ability to govern and control a pandemic health threat is closely associated with state fragility and, as such, governments in developed countries are expected to demonstrate both robust capacity and political will in preventing and controlling pandemic disease, while maintaining a high level of social cohesion and equality (Fourie, 2007). Governed by its institutional logic, every government's Covid‐19 responses and interventions reveal how it intends to shape national safety and stability during the crisis (Thornton and Ocasio, 2008). A government's policies and measures in preventing and controlling the spread of the pandemic hence reflect its underlying logics of actions and institutional rationalization. Research from New Zealand shows that when a government manages to control the spread of a pandemic, societal trust increases, since people have to work together as a society to overcome the disaster and as such they develop a deeper trust of the government's institutional logic (Sibley *et al*., 2020).

It is noticeable that across the globe, governments have responded to the threat of Covid‐19 with different measures. These include new communication campaigns to provide guidance and support, social distancing measures, national and/or regional lockdowns, tightening business and public organizations’ hygiene standards, etc. Some governments also developed policies and intervention schemes that offer financial support to employees and businesses, while others have focused on increasing healthcare capacity through hospital building and sourcing of personal protection equipment to increase supply.

Nevertheless, the nature of the Covid‐19 pandemic is boundary‐crossing. It crosses national and territorial boundaries and presents a global health problem beyond national borders. While each national government offers different advice and strategies to mitigate and control the spread of Covid‐19, the discrepancy and often conflicting approaches enacted by different governments present challenges to one particular vulnerable group of people – international migrants. As people who have moved across international borders from their original habitual place of residence to find work or better living conditions, international migrants tend to hold strong connections to home, and their behaviour and decisions are usually shaped by both home and host‐country institutional logics (Pio and Essers, 2014). Consumer behaviour studies often discuss international migrant behaviour and consumption choice from the lens of their culture and identity, positioning them as ‘ethnic’ consumers, discussing their acculturation process and outcomes (e.g. Askegaard, Arnould and Kjeldgaard, 2005; Peñaloza, 1994; Yu *et al*., 2019), and how their identity choice or conflict shapes their consumption practices (e.g. Cappellini and Yen, 2013, 2016; Yu *et al*., 2019).

Unfamiliar with host‐country institutional logics, migrants are likely to experience higher levels of anxiety, stress and other psychological trauma than non‐migrants when interpreting, responding and adapting to new Covid‐19 rules and measures imposed by the host‐country government (Kumar *et al*., 2020). Although migrants’ understanding of the values prevalent in the host country tend to increase over time (especially those who are eager to integrate into the host society) (Röder and Mühlau, 2012), many migrants are still inclined to refer to their home country's government advice in a crisis situation because it represents a more familiar and identity‐relevant frame of reference (Thornton, Ocasio and Lounsbury, 2012), regardless of whether it is dissimilar to or conflicting with their host country's advice, and reflecting a different set of institutional logics. Previous research shows that when people encounter different and contradictory institutional logics in consumption objects, they may perceive an incongruence between their identity project and their consumption choice, thereby experiencing identity conflict (Zanette and Scaraboto, 2019). Nevertheless, little research attention has explored how conflicting institutional logics embedded in consumption choice of practices would affect people's coping behaviour. To address this research gap, this paper explores migrants’ experiences during Covid‐19 and discusses how they navigate through dissimilar host and home government's advice and policy regulations during their coping.

## Methodology

### Research context

The UK has been badly affected by the spread of Covid‐19 and on 23 March 2020, the UK government imposed a strict national lockdown, banning all ‘non‐essential’ travel and contact with people outside one's home, while shutting all schools, businesses, venues and facilities (Picheta, 2020). People were told to stay at home, save lives and protect the NHS. While the general public were advised to practise social distancing and avoid gathering in public, the police were also empowered to enforce the lockdown. Although the government announced that the UK had passed the peak of its outbreak in late April, only in late June was the lockdown gradually eased. Table 2 details the measures imposed by the UK government during the data collection period (1 April–15 May 2020).

**Table 2:** UK Covid‐19 government lockdown measures

The UK Covid‐19 government lockdown measures (1 April–15 May)
Public to avoid leaving houses apart from shopping for basic necessities, such as food or medicine. Outings allowed for any medical need, to provide care or to help a vulnerable person. One form of exercise per day allowed, alone or with members of your household. Social distancing of two metres where possible, with one metre minimum for any outings. All gatherings of more than two people in public prohibited – excluding people you live with. All social events, including weddings, baptisms and other ceremonies cancelled, excluding funerals. School closures. Workplace closures if working from home is possible. Non‐essential businesses and industries closures, including clothing and electronic stores and other premises including libraries, playgrounds and outdoor gyms, and places of worship. Public event cancellation. Restriction on internal movement. International travel controls.

*Source*: GovUK (2020).

**Figure 1:**
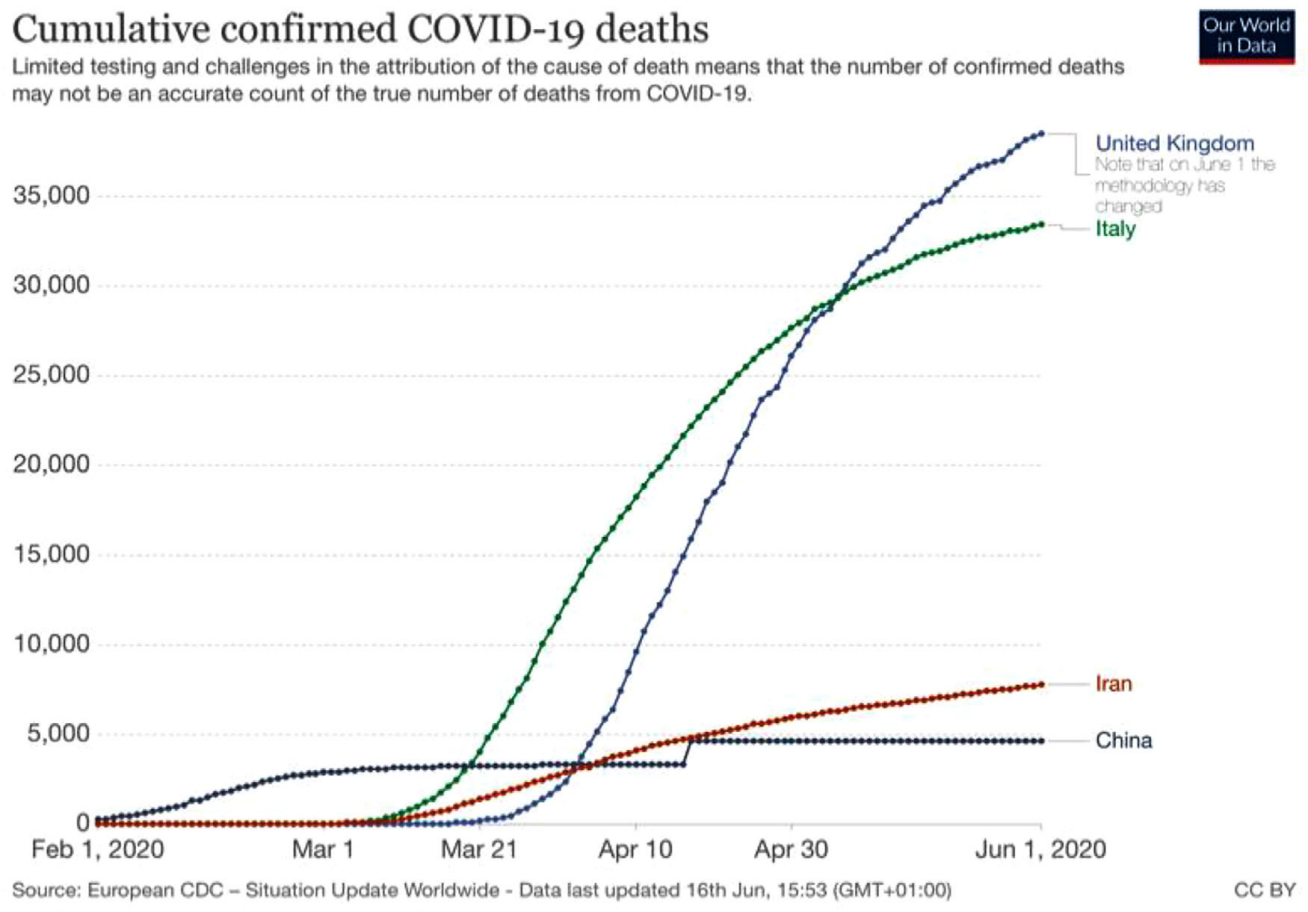
Covid‐19 deaths: linear chart in selected countries between 1 February and 1 June (ECDC, 2020) [Colour figure can be viewed at wileyonlinelibrary.com]

While the UK presents a suitable research context to study coping strategies, migrants from China, Italy and Iran were chosen to be interviewed as research participants because they were the top three countries worst hit by Covid‐19 in terms of death figures published by the European Centre for Disease Prevention and Control (ECDC) by the end of March. Figure 1 shows the Covid‐19 death progression within the same time period (ECDC, 2020), illustrating the contextual differences between the three countries, with the UK as the benchmark.

Table 3 describes the key measures and policies imposed by the Chinese, Italian and Iranian governments, compared with the UK, to prevent and control the spread of Covid‐19. In contrast, the UK lockdown rules were less strict, as the UK government did not encourage mask wearing for outings in April and May, and only changed its stance regarding mask wearing on 24 July.

**Table 3:** Lockdown measures comparison

	UK	China	Italy	Iran
Similarities	School closures Workplace closures if working from home is possible Non‐essential businesses and industries closures Public event cancellation Restriction on internal movement International travel controls			
Differences	‐ Public to avoid leaving houses apart from shopping for basic necessities, such as food or medicine ‐ One form of outdoor exercise allowed per day, alone or with members of your household ‐ Instructions to wash hands regularly ‐ Social distancing of minimum one metre for any outings, ideally two metres+ ‐ Mask wearing discouraged for the general public ‐ Mask wearing recommended for ill and vulnerable groups only, such as NHS staff and social care workers ‐ Lockdown easing from 15 June ‐ Compulsory mask wearing in public space starts on 24 July	‐ Compulsory mask wearing in public spaces ‐ In some areas, outings limited to one family member per household every two days to buy necessities ‐ Temperatures checked before entering public buildings ‐ Chinese officials going door to door for health checks, and forcing anyone ill into isolation ‐ Public transport closure in some areas ‐ Lockdown easing from 8 April in some provinces	‐ Compulsory mask wearing in public spaces ‐ Public to avoid leaving houses apart from shopping for basic necessities, such as food or medicine ‐ Outdoor exercise not permitted during lockdown ‐ Social distancing of one metre for any outings ‐ Lockdown easing from 3 June	‐ Public to avoid leaving houses apart from shopping for basic necessities, such as food or medicine ‐ Social distancing of one metre for any outings ‐ Mask wearing encouraged for outings; made compulsory on 8 June ‐ Reopening of low‐risk businesses and industries from 11 April ‐ Reopening of religious gatherings from 18 April ‐ Lockdown easing from 20 April

*Sources*: BBC News (2020a, 2020b); Beaumont (2020); GovUK (2020); Graham‐Harrison and Kuo (2020); Schofield (2020); Shahla (2020); Whelan (2020); WHO (2020).

### Recruitment and participants

An interpretivist paradigm guided this research to explore international migrants’ coping experiences during the UK lockdown. Recruitment was facilitated through the research team's personal contacts, with a view to identifying international migrants from China, Italy and Iran, who self‐identified as being emotionally affected and having adapted their behaviour in an attempt to cope with the Covid‐19 crisis in the UK. The first few participants from each country were identified through personal contacts and then a snowball sampling approach was applied via participant referrals. Eligibility criteria included being 18 years of age or older; having been in the UK for more than 1 year; being born in China, Italy or Iran; and having fluency in English, Mandarin Chinese, Italian or Persian. Second‐generation migrants were excluded, as their acculturation may be different (Hendy *et al*., 2019) and consequently they might have a different level of understanding of the UK government's institutional logics. To gain an in‐depth understanding of each migrant group's coping experience, 20 participants from each sampled country were recruited and interviewed (see full list in Appendix A in the online Supporting Information).

In total, 60 migrants participated in our research, including 20 from each of the sample countries. The gender distribution was fairly equal, consisting of 29 females and 31 males in total. The average age was around 39 years old, with the Chinese sample group being a few years older (m = 44) than the Italian (m = 37) and Iranian (m = 36) samples. All except one of the participants were based in England, and most of them were located in the Southeast of England. The participants had lived in the UK for an average of 11 years, which suggests that they are not new migrants and have been in the process of acculturation for quite some time. Table 4 provides a detailed summary of the participant profile across the three sample groups.

**Table 4:** Participant demography

	Total	Chinese (N = 20)	Italian (N = 20)	Iranian (N = 20)
Age: mean (range)	39 (21–67)	44 (26–58)	37 (29–55)	36 (21–67)
Gender	M: 48%, F: 52%	M: 50%, F: 50%	M: 50%, F: 50%	M: 45%, F: 55%
Average years in the UK (range)	11 years (1–34 years)	15 years (3.5–34 years)	8 years (3–10 years)	11 years (1–20 years)
Living with family	65%	80%	50%	65%
Have children	38%	65%	20%	30%
Main purposes of migration	Better work opportunities, higher education, better human and women's rights	Better work opportunities, higher education, better human rights	Better work opportunities	Better work opportunities, higher education, better women's rights
Work role examples	See subgroup breakdown	Engineers, company managers, entrepreneurs, academics	Technicians, van drivers, project managers, entrepreneurs, beauty therapists, consultants	Traders, accountants, data scientists, account managers, salesmen

### Data collection

Semi‐structured interviews were employed to understand how participants made sense of the government measures, the strategies they enacted to assist their coping and how their everyday life was affected by the lockdown, allowing extraction of meaning from migrants’ stories and experiences. Ethical approval to conduct this research was obtained prior to data collection from the lead author's institution. All participants were emailed or messaged a recruitment advert, a detailed information sheet and a consent form in English, explaining the purpose and reasons for the interviews and the research procedures.

All interviews were conducted online by a team of researchers who are fluent in Mandarin, Italian or Persian, using the mother tongue of respondents. Interviews started with general questions, such as personal background, profession and their motivation to move to the UK initially. Afterwards, the questions explored participants’ understanding of the lockdown and the overall governmental measures in their home country and in the UK, their daily experiences of mandatory confinement and their coping‐related consumption practices. The design of the interview questions covers multiple dimensions of the phenomena, concerning the impact of Covid‐19 on the migrants and their consumption practices. Table 5 lists the detailed interview questions. Each interview lasted approximately 30–40 minutes.

**Table 5:** Interview questions

Warm up	**What do you know about Covid‐19?** ‐ Where did you obtain this information (UK/home news, UK/home social media)? ‐ How has it affected you so far? Can you give us an example? ‐ How are you feeling?
Home institutional logic	**How is Covid‐19 in your home country (China/Iran/Italy)?** ‐ What do you think of the home government's approach? ‐ How does the (home) general public react regarding the virus? ‐ Do you agree with the (home) guidance/suggestion/practices?
Host institutional logic	**What do you think about the UK approach?** ‐ What do you think of the UK government's approach? ‐ How does the (UK) general public react regarding the virus? ‐ Do you agree with the (UK) guidance/suggestion/practices?
Similarities and differences	**Are there any similarities and differences between your home country and the UK's approach?** ‐ For example? ‐ What is the impact of these differences on you? What do you do? ‐ How do you feel about the differences?
Individual coping	**What are you doing to cope with the threat of Covid‐19?** ‐ Mask wearing/isolation/washing hands? ‐ How does Covid‐19 affect your shopping and consuming behaviours? What are they? Why? ‐ Have you adopted any new practices because of Covid‐19? What are they? Why?
Family coping	**How is your family (or household) coping with the virus in the UK?** ‐ Can you give me an example? Is this a home/UK/your own strategy? ‐ Where/how have you learnt this? ‐ Do you need to persuade other family members to follow the same strategy? Why?
Community coping	**How would you say fellow migrants in the UK are coping with the outbreak?** ‐ Is there a community feel? Do you know if they are taking on the home/UK/migrant community's own strategy? ‐ Have you noticed any community behaviour difference due to the virus outbreak? Can you give me an example?

### Data analysis

**Figure 2:**
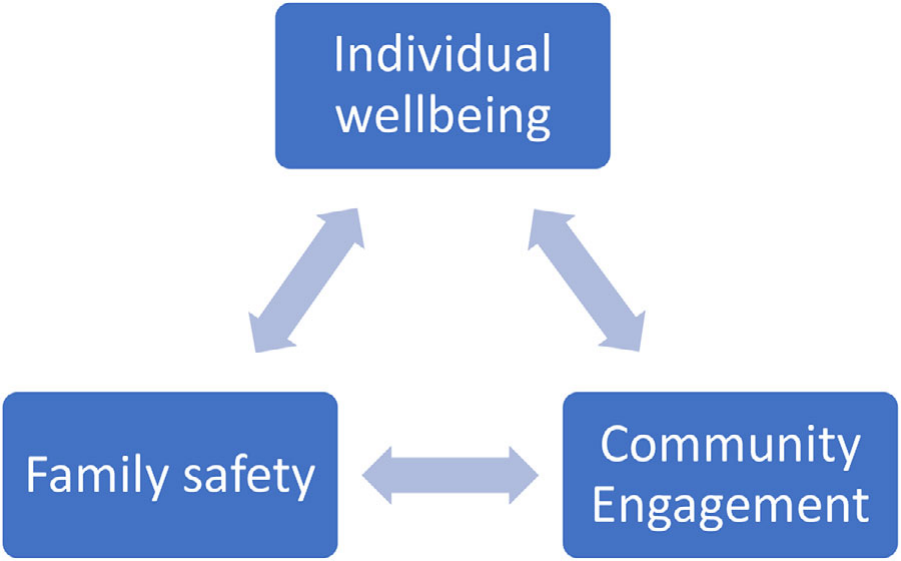
Analytic triangle [Colour figure can be viewed at wileyonlinelibrary.com]

The data analysis follows a step‐by‐step procedure to ensure rigour (Gioia, Corley and Hamilton, 2013). Table 6 describes the procedures, starting from data transcription and translation, initial manual analysis and the development of an analytic framework (Figure 2), to the progress of data structure charts (Figures 3a and 3b), including first‐order quotes, second‐order themes and aggregated dimensions (Ladge, Clair and Greenberg, 2012; Pratt, Rockmann and Kaufmann, 2006).

**Table 6:** Data analysis process

**Figure 4:**
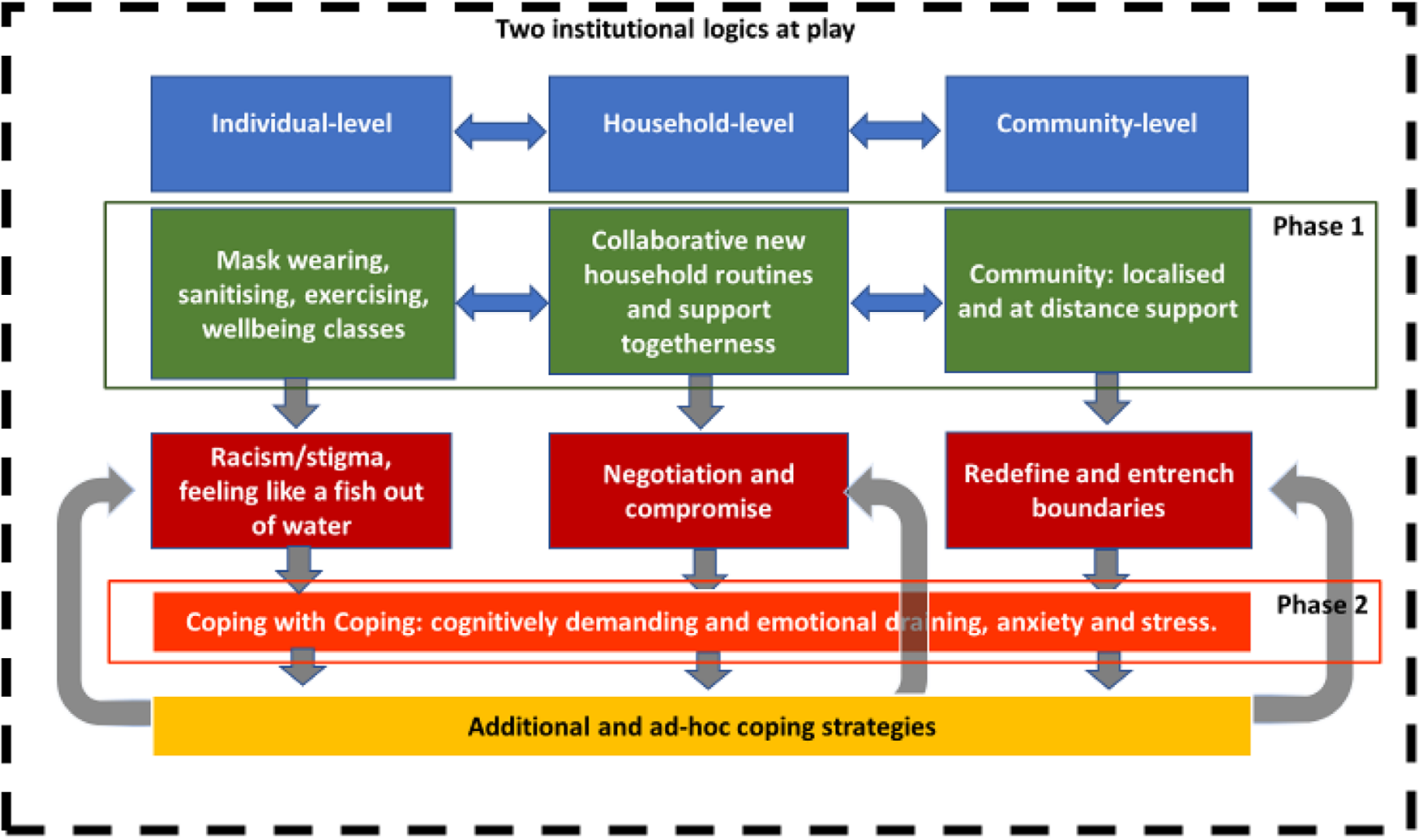
The model of coping with coping [Colour figure can be viewed at wileyonlinelibrary.com]

Data analysis process
Interviews were transcribed and translated into English by bilingual researchers, who conducted the interviews so that they could describe not only the words but also the emotions observed during interviews. Based on the first five interviews conducted from each migrant group, an initial manual analysis was conducted by two people separately, using an open coding technique to identify various coping strategies. The two researchers then systematically collated the notes by exploring individual interpretations to reach consensus, through an analysis of key coping strategies occurring at individual, family and community levels. This led to the development of the analytic triangle (Figure 2), which we used as a guide to later data collection and subsequent data analysis. After all the data collection and transcription was finished, we imported the 60 interviews into NVivo and used NVivo to help further develop first‐order (participant‐centric) codes and their associated quotes. Following the analytic triangle (Figure 2) and through the interpretation of the codes and quotes, we adopted a continuum back and forth between the emerging themes (individual wellbeing, family safety and community support) and the literature on coping, to organize first‐order concepts into second‐order (theory‐centric) themes and the aggregated dimensions. See Figures 3a and 3b, which provide demonstrations of the development of our coding progress. Then the research team discussed the static codes identified to develop and add three‐dimensional relational dynamics and intertwined movement, showing how the themes related to each other, leading to the origination of a paradoxical coping concept (see Figure 4).

Figure 3(a) Data structure chart A. (b) Data structure chart B [Colour figure can be viewed at wileyonlinelibrary.com]

**Figure:**
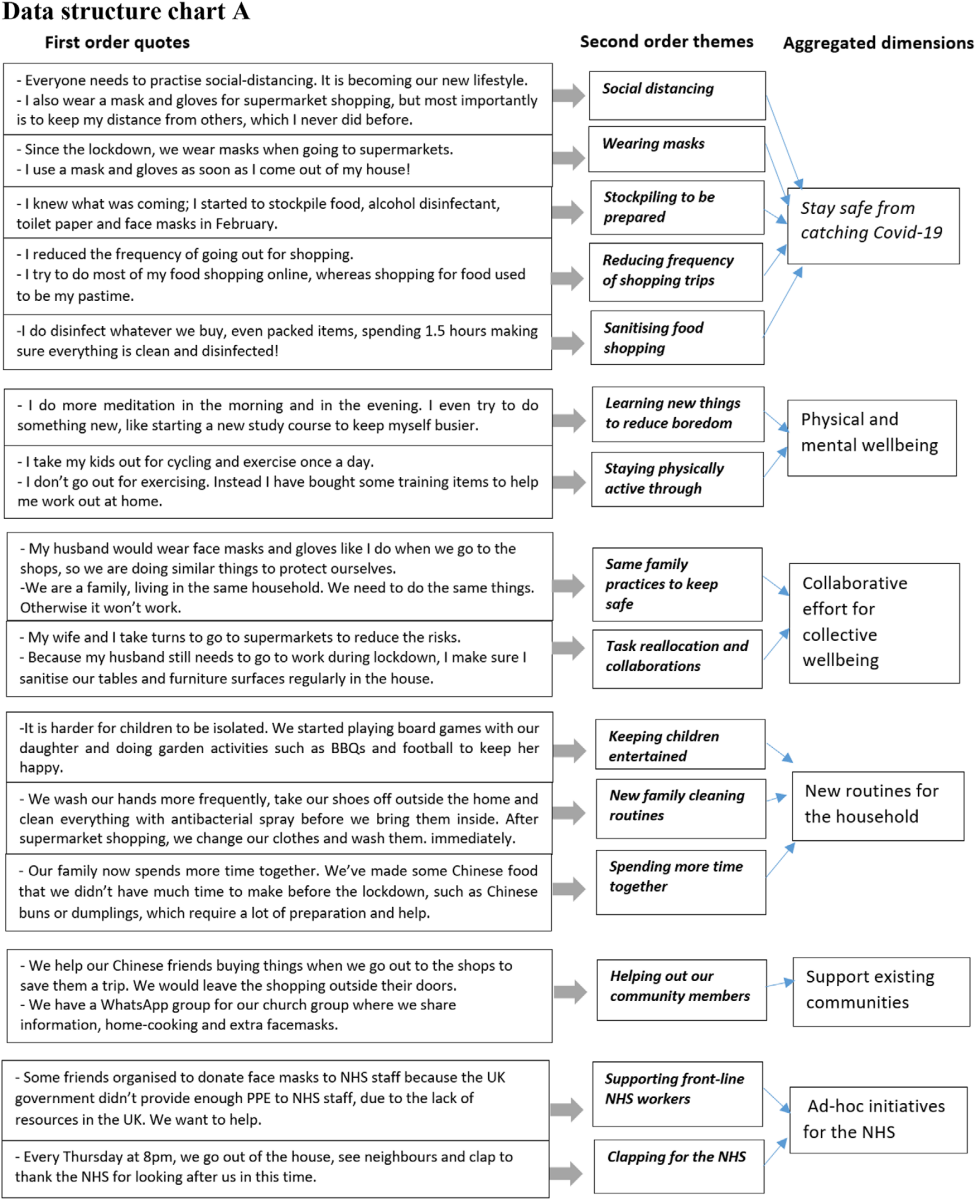

**Figure:**
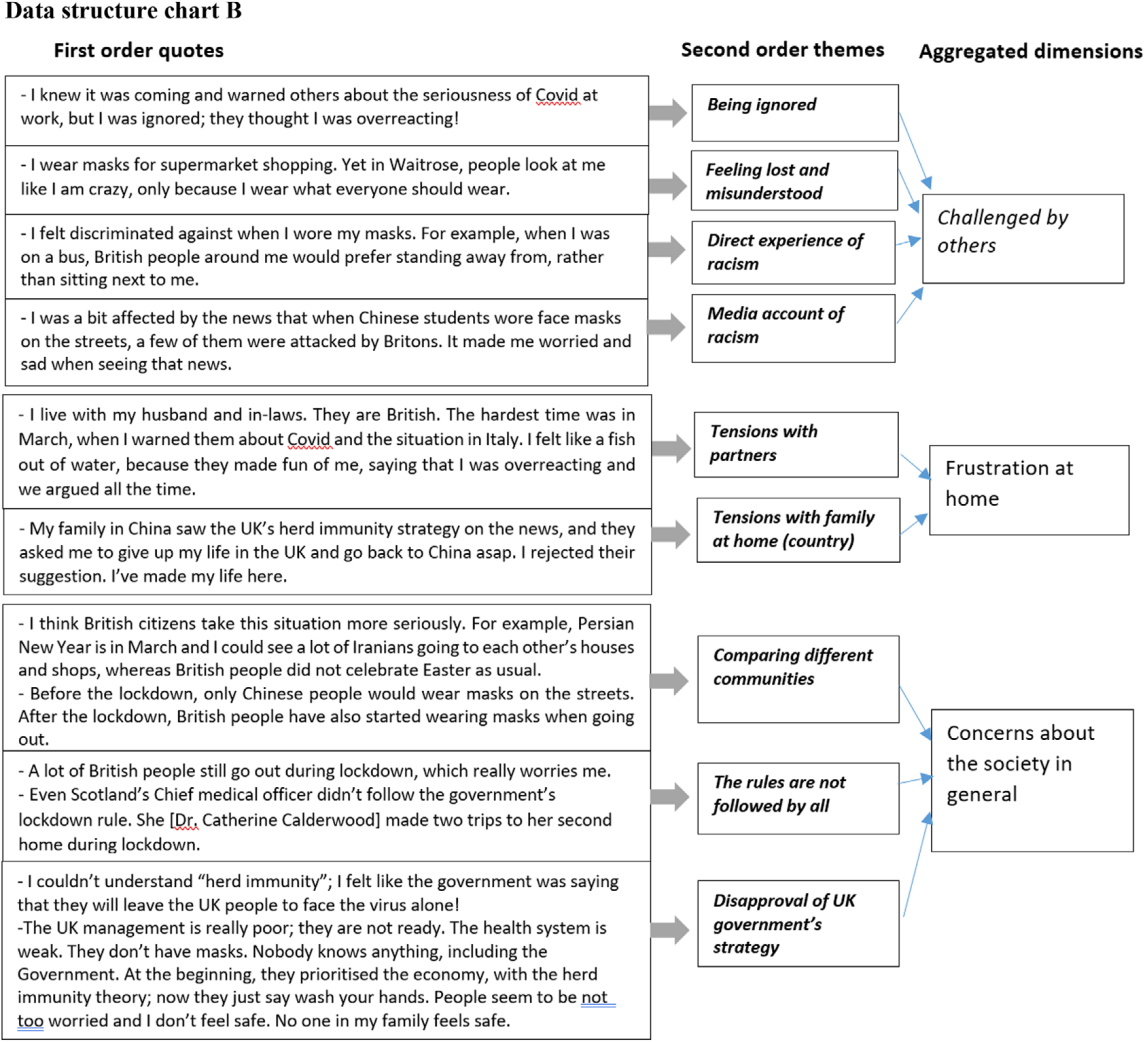

## Findings

Participants were well aware of the measures taken by the UK government during lockdown and were able to describe and comment on them in detail, showing how they could make sense of the actions of the institutions of the host country (Scott *et al*., 2000; Thornton and Ocasio, 1999). It is probably because of their knowledge of the measures of the UK government and the situation in their home countries, that participants’ anxiety and stress were described as more intense compared to their British counterparts:

We are more worried about the virus than the British, because we (Italian) have experienced it twice, once in Italy through parents and friends and then here! (Arianna – Italian)

Participants’ emotional reactions confirm Jin's (2009, 2010) studies showing how people tend to experience anxiety and fright when the situation is perceived as unpredictable and out of control. However, as we will see later, participants also described other emotions linked to their specific contexts. To cope with the stressful uncertainties due to their experience of the virus in both home and host countries, participants activated some behavioural strategies to ensure wellbeing and safety. Informed by our analytic framework (Figure 2), we present an overview of the shared and multi‐layered coping strategies at individual, household and community levels, as well as coping conflicts, with demonstrated evidence in Figures 3a and 3b.

### Individual strategies

While previous literature on health care and coping (Hutton, 2016; Leenerts, Teel and Pendleton, 2002) refers to self‐care activities with developmental aims, our participants described a series of self‐care strategies for two very different purposes: avoiding contracting the virus and circumventing being mentally affected by the lockdown. Both of these caring practices were planned via a mix and match of information, products and services acquired from the home and the host countries.

Mask wearing was certainly the most commonly mentioned consumption practice to protect their bodies from the virus. At the time of the fieldwork, the UK government had not changed its stance and was still advising against the benefits of wearing face masks to the general public (Boseley, 2020), but participants offered counterarguments, citing sources from their home countries, including newspaper articles and information received through networks of friends and family. Some were also actively involved in convincing sceptical British colleagues and friends via social media regarding the benefits of mask wearing. Nevertheless, despite sharing the same behaviour, Chinese participants were more cautious in choosing whether they should wear a mask or not:

My husband and I started wearing face masks when going on public transportation before the lockdown in the UK. We decided to do that as Chinese media said that some people might have no Covid‐19 symptoms, but they could still be contagious and spread the virus to others. We also asked our son to wear a face mask to school… However, he didn't want to do that because he didn't want to look different from other kids. We understand his concerns, so in the end we didn't push him to wear face masks to his school. (Don – Chinese)

Don reveals the complexity of adapting coping measures from the home to the host country. Aware of the increasing incidents related to hate crime, Don reflects on the difficulties of wearing masks and being at risk of racist attacks. Balancing out the risk of infection with the risk of being attacked was a family concern, and that is why Don and her husband consented to their son's request to not wear a mask to school. In her account there is an evident sensitivity of her son's vulnerability, which shows how behavioural coping strategies also have emotional dimensions for the self and for others (Cappellini, Marilli and Parsons, 2014; Duhachek and Kelting, 2009; Han, Duhachek and Rucker, 2015; Hutton, 2016; Kolier, 1991).

The acquisition of food was one of the most cited practices as a source of anxiety and stress (Hamilton, 2012). Nearly all the Chinese migrants interviewed stockpiled extra food, such as rice, noodles, frozen food and tinned food, with the intention of reducing the frequency of going out and ensuring that they had all the basics in the house during lockdown. Whilst the Italian and Iranian migrants attempted to reduce the frequency of their grocery shopping to around once a week, online grocery shopping was very much employed by Chinese migrants as an alternative.

When food shopping could not be avoided, participants described how such a mundane practice was performed with careful planning, aimed at protecting the body from the virus. As participants were aware that the virus could be transmitted via objects, any item entering the home was subject to a newly and carefully planned examination. While this newly established practice was carried out by one person in the household, it required the full collaboration of the family. Participants described how they had transformed areas of their houses into decontamination spaces, where they stored shopping and clothes for a certain amount of time before accessing them. Others, like Arezou, talked about the new cleaning practices which were adopted at home:

When I do any shopping, I make sure I leave the items outside the house, in the garage, and I bring the items in one by one. I disinfect all the items individually to protect my family. In terms of hygiene, it now takes a lot of time to sort out the shopping. I spend one and a half hours making sure everything is clean and disinfected… I learnt this through our Iranian community as people are sharing on Facebook their own way of dealing with different situations. I think this is more of an Iranian way, as I personally think British people are not very fussy about cleaning and disinfecting items. (Arezou – Iranian)

Having learnt from her Iranian community how to wash items purchased from the supermarket, Arezou described a new lengthy process that involves cleaning, storing and disinfecting shopping items before taking them into the house. Cleaning the home differently using new brands and products was also mentioned by many other participants, who admitted to washing door handles and other areas that were not usually cleaned.

If cleaning and using masks were the most commonly used health strategies for body protection, there were also new practices aimed at increasing wellbeing via alleviation of stress and diversion (Rückholdt *et al*., 2019). For example, the increasing domestic time was invested in learning new skills, including playing an instrument or learning a new language. For instance, Sadik admitted enrolling in a new online course to cope with the increasing boredom of spending unstructured time indoors:

I try to do something new and I am starting a new study course to keep myself busier… (Sadik – Iranian)

Engaging with daily exercises at home was described as a new routine, established to avoid having to go out, thus reducing the chances of catching the disease:

I can exercise each morning and also follow YouTube clips doing exercise in the afternoon, such as weight training, yoga, aerobic or anaerobic exercise. My husband and I are active and we enjoy sports. We are sad that our gyms are closed during lockdown. (Aimee – Chinese)

Readapting the practice of exercising from the gym to the domestic environment was seen as a compromise that some participants embraced with the acquisition of new equipment and consumption of videos. These newly established practices to increase wellbeing show how, despite the adversities of the mandatory confinement, participants create occasions for personal development, reframing their routines and domestic life in experiences of self‐development, because creating occasions of emotional and experiential self‐protection are beneficial for managing stressful situations (De Terte, Stephens and Huddleston, 2014; Pavia and Mason, 2004).

### Household‐based strategies

While individual strategies were clearly illustrated in participants’ narratives, their practices were not carried out in isolation, but performed with household members, showing a relational dimension, since care for the self has an impact on others living in the household (Cappellini, Marilli and Parsons, 2014; DeLongis and Holtzman, 2005; Hutton, 2016). However, there were also coping strategies that were aimed at the entire household, planned and activated for their collective safety and wellbeing. Establishing new routines and standards was seen as a collective effort, which, although carried out by one member of the family more than others, required the commitment and collaboration of all members. For example, Mahin explains how shopping is his task while storing food is his wife's responsibility:

I live with my wife and our girl… I try to do the food shopping now to protect them, as I don't want my wife to go out with my girl… but as soon as I get home, she takes over the shopping and starts disinfecting everything. I even clean my shoes with alcohol. All the door handles are cleaned many times a day… (Mahin – Iranian)

Interestingly, Mahin describes how the mundane practice of shopping has now been divided into new and different tasks (cleaning door handles, cleaning shoes), with specific responsibilities for himself and his wife. This description of sharing tasks and responsibilities collectively echoes previous literature showing how the collective enactment of coping strategies proved beneficial for the household (Baker, Gentry and Rittenburg, 2005), and hence reduced the level of stress and anxiety of individuals and the family. The introduction of new cleaning practices and routines, aimed at protecting the health of the household, was planned, together with the redefinition of leisure activities in the domestic setting. As such, a mundane practice like cooking, previously performed by a family member, became a collective practice (see Figure 3a). In particular, preparing and making home (country) dishes became a way of spending family time together. There were also other activities, organized by parents, which required the acquisition of new equipment, including board games, consoles and gadgets for the garden. In our case, participants often referred to new resources acquired from both home and host marketplaces as a way of re‐establishing a sense of ‘normality’ at home, with the consumption of new board games, cooking ingredients and technology.

### Community‐based strategies

Besides family and immediate others, participants also mentioned a network of relationships outside their domestic setting. In particular, they refer to existing communities which were present in their lives before the lockdown. As such, we did not witness any new network being formed as a coping response to the crisis, but we witnessed an intensification of existing networks through information and resource exchanges. Interestingly, Chinese participants were particularly vocal in describing the solidarity of their communities, showing how migrants tend to emphasize the positive achievements of their own ethnic groups when feeling discriminated against (Moroşanu and Fox, ; Rzepnikowska, 2019). Firstly, participants seemed connected to fellow Chinese migrants through various social media platforms to exchange and share practical information, provide emotional support and stay updated about the development of the pandemic crisis:

I think Chinese people in the UK are happy to help each other during lockdown if anyone needs help. There is a sense of community among Chinese people living in the UK. For example, we encourage each other to keep fit and well, and some people provide Chinese medicine tips to others, which might boost the immune system. The community feeling gives us a sense of belonging. (Oren – Chinese)

If Oren talks about an undetermined ‘community among Chinese people’ in which material and emotional help is provided via tips on how to keep fit (physically and mentally), there are also examples of localized communities where material support is more prominent. More localized communities were also described by other participants, like Zabi, who talks about a WhatsApp group that she has created with her colleagues to exchange recommendations on where to acquire food items that are in short supply:

I have a chat group with my colleagues, and we help each other with regard to what to buy and what to do. For example, at the beginning of this situation, when there was a shortage of toilet paper, eggs and rice, we used to let each other know where to go and buy them. (Zabi – Iranian)

Participants are particularly supportive of these localized networks providing ‘a sense of belonging’, which in a time of crisis seems to be crucial to cope with daily adversities (Marlowe, 2015). In this case, the sense of belonging is built around pre‐existing identity connotations related to work, ethnicity or simply geographical proximity. Interestingly, other identity connotations which the literature has shown as being fundamental before crises, including life stage, family roles and consumption interests (Kleine, Kleine and Ewing, 2017), seem to be less prominent for constituting a sense of belonging. Participants also referred to initiatives organized *ad hoc* with fellow migrants to support local hospitals and the NHS via symbolic practices (clapping together with neighbours), but also donations and material support (providing masks and meals to hospitals). Participants’ accounts of their support for the NHS (see Figure 3a) suggest that some participants were involved in many informal and spontaneous initiatives where their membership of various communities clearly emerged. This shows the complex network in which some of our participants are embedded, and the support they can receive as well as provide to their fellow members.

### Coping with the conflicting context

The aforementioned coping strategies are inserted in a context that is not always supportive but can be openly hostile to migrants and their behavioural changes. Figure 3b illustrates the conflict encountered by participants at individual, family and community levels.

At an individual level, participants talk about the difficulties encountered in their work environment before the lockdown was announced, and how they coped with the unexpected challenges faced with critical colleagues, friends and the general public. Wearing a mask caused stress and anxiety, as participants described feelings of being misunderstood or ignored:

It has been very disheartening and frustrating being ignored. Before the lockdown, I experienced a racist episode in the supermarket. An old man challenged me; he shouted and he spat right back at me saying ‘we are not dying’, only because I was wearing a mask. I was in a state of shock all day. (Alessandro – Italian)

While following the lockdown measures of their home country made participants like Alessandro feel ‘safer’ and more self‐confident, the unexpected negative reactions from the local environment triggered unexpected emotional stress and anxieties. To cope with such unexpected hostile and discriminatory reactions (Berjot and Gillet, 2011), which participants perceived as unjust and racist, they enacted additional coping strategies at individual, household and community levels (see Table 7). At individual levels, participants continued to follow home‐country guidelines, which were judged more adequate, even at the risk of being criticized. Others decided to withdraw from consuming media from the home and host country, and a few took more drastic decision and planned to relocate to the home country, since the hostile environment was seen as simply ‘too much to cope with’. There were also some cases in which participants decided to stop following their home‐country guidelines, even if regarded as more appropriate:

**Table 7:** Additional coping strategies

Additional individual coping strategies
Ad‐hoc strategies *Even though I was advised to wear masks, I chose not to wear masks a couple of times when I was on the public transportation. I did not want to draw the unnecessary attention to myself*. (Chinwen – Chinese) Ignore the context and focus on my own practices *The discrimination towards Chinese people in the UK worries me. My husband and I think some British people were irrational when linking the virus to Chinese people living in the UK. However, I cannot do anything about it, so I guess I only need to focus on protecting myself from the virus and take other possible risks…* (Aimee – Chinese) ‐ Disregard the UK guidelines and only follow home‐country guidelines *I've decided to follow the Italian guidelines, I am very worried about the situation in London*. (Alessandro – Italian) ‐ Disengaged with news for a temporary escape *I used to watch Italian TV and BBC news, but recently I have been avoiding it because it causes me stress and anxiety*. (Daniela – Italian) Plan to relocate to the home country *I am very disappointed and I don't feel safe. I feeI frustrated. I shouldn't say this, but I don't trust the UK government now. I would love to go back to my Italy*. (Letizia – Italian) Stay positive and be grateful *It is mind boggling that some people do not seem to understand the seriousness of this virus and are continuing with their everyday lifestyle as before, which puts people at risk. But I remind myself to be grateful that I live in a society where human rights are given to its citizens*. (Maeda – Iranian)


Additional family coping strategies
Negotiations and compromises *I asked my fiancé to follow my practices, such as wearing masks and gloves, so he can better protect himself. He didn't want to wear face masks or gloves before, because he didn't think they are needed. I sent him many links of articles to explain the importance. We argued… now he does it now because of me*. (Haiyang – Chinese) Divide family space to create distance *It's really hard, because my partner and I have to spend all time living in the same house. In a normal life, we would spend only three hours together but now all day. We want to see each other as less as possible. Fortunately, we live in a big house with a garden. We spend time in different rooms during the day*. (Letizia – Italian) Family relocation to another country *I don't feel safe, this is why I go out as less as possible. My husband and I will leave the UK after the lockdown, we will take our baby daughter with us to go to France*. (Daniela – Italian)


Additional community coping strategies
Strengthening the migrant community support to each other *Majority of Italian people that live here, have called me. We did not use to call each other that frequently. There is a greater sense of community in crisis, we help each other because this is not our country*. (Arianna – Italian)*I think there is a sense of solidarity among Italians in London. Where I live, there is huge community. We are easily demoralized compared to British and this kind of support is needed for our community*. (Marta – Italian)

When I was at work, my students and colleagues stood very close to me when they interacted with me; I did not feel comfortable. I didn't wear face masks though until the lockdown announcement because I didn't want to look different from others. I worried about other people's reactions when seeing me wear face masks. (Eming – Chinese)

While Eming acknowledged the importance of using a face mask outside, she admitted stopping using them for fear of ‘looking different’. As she admitted, this was not an ‘ideal solution’ but in evaluating the various possibilities of protecting herself or looking different, she decided to ‘blend in’. Such a strategy created discomfort and worries about personal safety which she decided not to declare to colleagues. These were strategies enacted to decrease the importance of their own identity as migrants in defining their coping strategies, while continuing to emphasize their identity as migrants in other aspects of their daily lives (Berjot and Gillet, 2011). Intriguingly, participants admitted that adopting compromised strategies to cope with a hostile environment (i.e. stop wearing a mask) created additional anxieties and stress, since they openly contradicted their initial plans of self‐protection.

If participants admitted feeling misunderstood and ignored at work, they also expressed frustration and discomfort at home. Conflicts on how to organize cleaning in the household, what to wear outside and how to organize food shopping were common in the narratives of intercultural couples. Lucia expressed the emotional conflicts in her household, which she shares with her husband and in‐laws:

My [British] husband and my in‐laws are more concerned than before. Perhaps it's because of my presence here. At the moment, I live with them. At the beginning, I felt like a fish out of water, because they made fun of me, saying that I was overreacting; now they have understood that Covid‐19 is a serious issue. (Lucia – Italian)

Feeling like a ‘fish out of water’, as Lucia says, was a common experience for many participants, admitting how their behaviour, which was aligned with the guidelines and norms of their home countries, clashed with the behaviour of their loved ones or people who they shared a household with. Such a clash caused day‐to‐day conflicts, but mostly an overall sense of ‘being different’ and marginalized, or even ridiculed.

Some participants also acknowledged tensions which occurred with family from their home (country). Aimee mentioned how the conflict that occurred between her (British) husband and her (Chinese) parents upset her:

My husband is British, so his coping strategies are a bit different compared to mine. For example, he didn't want to wear masks, and thought wearing masks is very troublesome and uncomfortable. However, my parents in China insisted that we wear masks. My husband didn't want to listen to my parents at the beginning, and I was really upset about it. (Aimee – Chinese)

To cope with the additional stress caused by a domestic hostile environment, participants enacted strategies of selective affiliation (Berjot and Gillet, 2011), consisting of reducing the time spent with other family members and avoiding shared domestic spaces. Others admitted engaging in heated debates in an attempt to negotiate and compromise, which was emotionally draining. In a few cases, such tensions escalated to such an extent that they considered relocating to another country as a family.

Conflicts at a community level seem to be less prominent with Chinese and Italian participants, while Iranian participants took a less favourable view in comparing their own communities with the British population:

I feel that a large majority of Iranians in the UK do not seem to fully understand what social distancing or self‐isolating means. For example, they feel going to the park for a picnic with their family and friends is acceptable, as they all know each other. This is the completely wrong approach to this pandemic, and I feel that this is mainly because they only follow Iranian channels and are unaware of the guidelines in place by the UK government. (Maeda – Iranian)

Isolation and lack of integration into British society are cited by Maeda as causes of Iranians disregarding UK guidelines. Other participants admitted that cultural celebrations planned just before the lockdown, including Persian New Year, also posed difficulties amongst the Iranian community. The narratives echo previous studies (Moroşanu and Fox, ; Rzepnikowska, 2019) showing how migrants can be overcritical of fellow migrants in an attempt to dissociate themselves and destigmatize their own identity.

Chinese and Italian participants seem to be included in more active communities, where criticism towards episodes of racism and the overall actions of the UK government is more prominent. Many of them were worried at the loose enforcement of lockdown rules and disappointed with the UK government's overall approach in managing the pandemic (see Figure 3b). To cope with the stress and uncertainties caused by the hostile environment, some participants adopted a strategy of re‐evaluating some dimensions of their own collective identities and reframing them as positive instead of negative (Berjot and Gillet, 2011). This was often linked with engaging with activities organized and enacted by local and national communities. Others decided to cope with the additional stress by enacting avoidant activities (Rückholdt *et al*., 2019), including disengaging with news and social media.

## Discussion

Our findings contribute to the interdisciplinary literature on coping by showing how coping is a multi‐layered and multi‐phase process, which we call *coping with coping*. As illustrated in Figure 4, coping is a process in which individual, household and community‐based coping strategies are included in an environment which can trigger additional anxieties, requiring individuals to enact new multi‐layered coping strategies.

The process starts with migrants’ enactment of a carefully planned, multi‐layered and contextually based set of coping strategies to navigate through complex and often conflicting government institutional logics that are imposed upon them (phase one). Findings show how initial coping strategies were enacted to solve anxieties caused by the crisis. Findings confirm previous works (Baker and Baker, 2016; Bruce and Banister, 2019) showing that individual coping strategies are relational because coping for the self is in relation to household members, neighbours and distant others (from the community of migrants in the UK, to the family in the home country). In fact, there are constant interactions and negotiations of coping strategies between the individual, household and community levels. As such, domestic routines would have been redefined, mundane practices (shopping) reorganized and responsibilities (cleaning and sanitizing) redistributed, echoing the work of Hennekam and Shymko (2020) regarding people's pragmatic adoption of various behavioural strategies.

Participants also discussed how they planned and procured extra goods in their shopping to help their community members, demonstrating multi‐layered involvedness. While previous works on communities of coping are often conducted in the work environment (Jiang and Korczynski, 2016; Tervonen and Enache, 2017), our findings reveal that during a crisis situation, collective coping strategies at the community level are also enacted outside of work. In contributing to the growing number of studies advocating for a contextual and collective understanding of coping (Bruce and Banister, 2019; Szmigin *et al*., 2020), we show how complex coping strategies materialize via consumer practices, where individual aims are intertwined with collective ones.

While the multi‐layered level of coping strategies might be applicable to all individuals facing a crisis, participants reveal the peculiarity of their strategies, which are a combination of practices from both home and host countries, informed by two different institutional logics (Cappellini and Yen, 2013; Yu *et al*., 2019). These practices are learnt, discussed and appropriated with family and friends from home, and colleagues, family, friends and fellow migrants in the UK, reflecting the two frames of reference (Pio and Essers, 2014; Thornton, Ocasio and Lounsbury, 2012). Having access to news and recommendations from a variety of sources, our participants were well‐informed and able to critically elaborate their domestic strategies, while acknowledging the crucial role of the institutional context, where they see themselves as migrants.

Nevertheless, although all participants engaged in various coping strategies through the acquisition of new practices and products available in the UK, there are national differences in their evaluation of the government's strategies. While our small samples do not allow us to provide any generalization, some differences between the three migrant groups are noteworthy. In our samples, Italian and Chinese participants are more critical of the UK government's lack of support in securing face masks, while Chinese participants are more active in planning collective initiatives to support each other. Even though some practices are adopted by all, migrants’ attitudes towards the same behaviour differ across various national backgrounds.

At an emotional level, the enactment of coping strategies in phase one caused additional anxiety, fear and sadness. Some participants had to justify their behaviour to relevant others, as well as readapt their strategies for fear of being discriminated against. This finding extends previous works (Jin, 2009, 2010) looking at the emotional dimensions of coping, since it shows how interactions with others may add new emotions during their enactment of coping. While Hutton (2016) shows that being able to cope helps individuals develop their wellbeing through increased resilience, the hostile environment does not necessarily increase migrants’ resilience, as it imposes a complex and demanding coping dilemma, where migrants are battling through both home and host sets of different institutional logics while coping with Covid‐19.

Paradoxically, the initial coping behaviour strategies, enacted to solve uncertainty and eliminate stress derived from an unpredictable crisis, often led to unexpected new anxiety and stress to migrants. In phase one, migrants experienced isolation, misunderstanding and at times racism, which created new anxieties and stress that they needed to cope with by adopting additional strategies (e.g. stop wearing masks, stop going out) that they were not entirely comfortable with. These additional strategies were not a direct response to the crisis, but an attempt to manage anxieties provoked by a hostile environment. They expose, rather than alleviate, migrants’ vulnerability to the original stressor (the virus). We refer to this as a coping paradox, where coping strategies enacted in a hostile host environment trigger new anxieties and stress that are not related to the original cause of stress and cannot be resolved by their initial coping solutions.

The presence of additional strategies enacted in phase two shows how coping is not a mono‐phase process, as conceptualized in previous literature, but rather a multi‐phase process which evolves by considering the contexts in which coping happens. The existence of a multi‐phase process contributes to the existing literature on coping and migrants (Mak and Nesdale, 2001; Moroşanu and Fox, ; Rzepnikowska, 2019). It does so by showing the importance of looking at how migrants’ coping strategies are perceived by the host country and how migrants respond to the hostile local environment, enacting additional coping strategies aimed at managing perceived hostilities including racism, but also tensions within the households. Our findings show that coping with coping is cognitively demanding and emotionally draining. Not being able to cope is frustrating, but not being able to cope righteously in ways that are deemed appropriate in one's evaluation is more stressful, as it causes cognitive dissonance and compromising actions. Even though additional as well as ad‐hoc coping strategies are exercised, they are only compromising solutions because they expose migrants’ vulnerability to the original stressor and reinforce migrants’ powerless position as marginalized citizens.

## Conclusion and implications

In investigating how international migrants in the UK cope with Covid‐19 and the government lockdown measures, this study contributes to the interdisciplinary debates on coping in various ways. In introducing the concept of *coping with coping*, it shows how coping is a multi‐layered and multi‐phase process, where migrants’ coping strategies are affected by the local environment, which is often hostile. Table 8 details how local and national initiatives can be tailored to support migrants during the various phases of the coping with coping process.

**Table 8:** Government policy recommendations

Individual migrants	**Use a variety of communication channels**. Migrants have their own social media platform preference; thus, the UK government is advised to utilize a variety of communication channels, including different social media platforms. This is because traditional channels commonly used to communicate information about public health, such as TV, radio or letters, are often ineffective in reaching migrants and other vulnerable residents, who might rely on alternative social media platforms, such as WhatsApp, WeChat or migrant‐owned newspapers that cater to those groups. **Language support** can help migrants overcome their potential language barriers and better comprehend the information disseminated by the UK government. As such, we would advise the government to ensure that all Covid‐19 communication campaigns are translated into various languages so that migrants are informed about and updated with the latest government guidelines.
Migrant families	**Offer local community support** to provide a safety net to migrant families, signposting local resources available and initiatives that migrants could participate in, in order to integrate into the community. **Provide social and emotional support to migrant families and acknowledge their problems in policy responses**. Migrants can suffer from job loss, income reduction, feelings of isolation and relationship breakdown following lockdown. The UK government could collaborate with migrants’ home governments to provide Covid funding schemes and the needed support to migrant families.
Migrant communities	**Work with migrant opinion leaders** to promote Covid‐19 prevention and control campaigns and obtain agreement from the migrants. Migrant opinion leaders can also help the government understand how migrants comply with its Covid‐19 policy measures and how these interventions are conformed to in specific contexts. **Promote societal harmony through social marketing campaigns** that identify and discuss all types of practices available and acknowledge their legitimacy. This helps increase citizens’ awareness and respect for the coping strategies of others, which promotes a less hostile environment to migrants who are keen to practise coping strategies acquired from their home country. It will also help reduce hate crime, stigma or racist behaviour associated with specific coping practices (e.g. mask wearing). **Work closely with journalists, media and non‐profit organizations (NGOs) to discuss migrants’ contributions to the combat of Covid‐19** to portray migrants in a positive light and combat disinformation that accuses migrants of spreading Covid‐19, not complying with lockdown rules, stealing jobs from locals or accessing free NHS treatments. Showing that migrants are very cautious in preventing and protecting themselves from Covid‐19 would help to protect migrants from stigmatization or hate crime.
National	**Better knowledge exchange and learn from other countries’ experience**. The UK government is advised to take a more proactive role in learning from other countries’ experiences and adopting best practices proven successful in countries that successfully controlled the spread of the pandemic. By showing their willingness to listen, understand and learn from other countries, it can help improve migrants’ confidence in the UK government and its favourability as a migrant destination. **Work with migrants to improve national brand image**. With its high death figure, international migrants now regard the UK less favourably as a possible migration destination. However, this could be improved by working closely with migrants, migrant leaders, migrant families and communities to increase migrants’ attachment and identification with the UK as a host country. This will lead to more positive word‐of‐mouth regarding the UK as a migration destination.

## Limitations and future research directions

As this is the first attempt to theorize the process of coping with coping, our study has some limitations. Firstly, although the findings discuss coping strategies at individual, family and community levels, the interviews were conducted with individual migrants only. Future research is therefore recommended to conduct interviews with other family members and members of the same migrant communities to obtain a collective perspective on family and community‐level coping strategies. Acknowledging the views of migrant community leaders and local policymakers responsible for migrants’ wellbeing can provide a more holistic picture and aid in policy recommendation.

Secondly, the majority of the participants in this research represent a group of professional migrants from relatively well‐off economic backgrounds, living in a developed country, despite their vulnerabilities and marginalized conditions. Future studies are advised to research migrants in low and middle‐income countries (LMIC) and consider related issues such as human rights and human dignity, and gender‐based abuse or violence, while pushing the boundaries of our current knowledge to develop resilience frameworks, useful to policymakers. Finally, further studies are welcome to investigate if, and if so how, the coping with coping process can be applied to other marginalized groups including, for example, BAME groups which have been affected by a hostile environment during the current pandemic (Crockett and Grier, 2021).

## Supporting information

Supporting Information
