# Supplementary material for: Coping with Coping: International Migrants’ Experiences of the Covid‐19 Lockdown in the UK
Source: British Journal of Management. 2021 May 25;32(4):1219–41. doi: 10.1111/1467-8551.12512 (PMC8209924; doi:10.1111/1467-8551.12512)
Supplement: Supplementary file 1 — Supporting Information [file BJOM-32-1219-s001.docx]

Appendix A: Participant list

| **Nationality and Name** | **Age** | **Gender** | **Location** | **Occupation** | **Number of years in the UK** | **Living with family** |
| --- | --- | --- | --- | --- | --- | --- |
| Chinese Aimee | 20s | F | Coventry | HR Personnel | 3.5 years | Yes |
| Chinese Baimei | 50s | F | Kenilworth | In retirement (was an engineer) | 34 years | Yes |
| Chinese Chingwen | 50s | F | Coventry | Entrepreneur (trading) | 20 years | No |
| Chinese  Don | 40s | F | Balsall Common | Housewife | 5 years | Yes |
| Chinese Eming | 40s | F | Coventry | Chinese teacher | 18 years | Yes |
| Chinese Finren | 50s | F | Reading | In retirement (was an engineer) | 34 years | Yes |
| Chinese Gouyan | 30s | F | Cardiff | Academic | 9 years | No |
| Chinese Haiyang | 30s | F | Coventry | Academic | 6 years | Yes |
| Chinese  I-ren | 30s | F | Rugby | Academic | 17 years | Yes |
| Chinese  Joug | 50s | F | Kenilworth | Chinese doctor | 12 years | Yes |
| Chinese  Keelong | 30s | M | Milton Keynes | Software engineer | 9 years | No |
| Chinese  Li | 50s | M | Leamington Spa | Academic | 10 years | Yes |
| Chinese  Mengren | 40s | M | Warwick | Entrepreneur (car) | 25 years | Yes |
| Chinese  Niwan | 30s | M | Nuneaton | Factory team leader | 12 years | No |
| Chinese  Oren | 30s | M | Colchester | Academic | 13 years | No |
| Chinese  Ping | 50s | M | North London | Entrepreneur (training) | 20 years | Yes |
| Chinese  Qui | 50s | M | Exeter | Principle researcher | 31 years | Yes |
| Chinese  Renfeng | 30s | M | Coventry | Automatic engineer | 11 years | Yes |
| Chinese  Suiren | 40s | M | Warwick | Policy manager | 17 years | Yes |
| Chinese  Tuzhen | 40s | M | Cambridge | Software engineer | 12 years | Yes |
| Italian  Roberto | 30s | M | East London | Moving light technician | 3 years | No |
| Italian  Marco | 30s | M | Central London | Sales agent supervisor | 6 years | Yes |
| Italian  Alessandro | 30s | M | Central London | Architect | 8 years | Yes |
| Italian  Giulio | 30s | M | Central London | Senior consultant | 7 years | Yes |
| Italian  Lorenzo | 50s | M | Essex | Mechanical engineer | 7 years | Yes |
| Italian  Paolo | 20s | M | Edinburgh | Amazon driver | 9 years | No |
| Italian  Marta | 30s | F | Chelsea | Lawyer | 4 years | No |
| Italian  Stefania | 30s | F | Marylebone | Director | 6 years | No |
| Italian  Agnese | 40s | M | Cambridge | Head engineer | 10 years | No |
| Italian  Rebecca | 50s | M | Oxford | Consultant | 20 years | Yes |
| Italian  Lucia | 30s | F | Stamford | English teacher | 5 years | Yes |
| Italian  Martina | 40s | F | South East London | Entrepreneur | 14 years | No |
| Italian  Simone | 30s | M | South London | Senior marketing manager | 7 years | Yes |
| Italian  Daniela | 30s | F | North London | Housewife | 4 years | Yes |
| Italian  Silvia | 30s | F | West London | Marketing manager | 6 years | Yes |
| Italian  Tommaso | 40s | M | Essex | Healthcare assistant | 9 years | No |
| Italian  Arianna | 20s | F | North London | Couture dressmaker | 7 years | No |
| Italian  Barbara | 30s | F | Birmingham | Project manager | 11 years | No |
| Italian  Letizia | 50s | F | West London | Photographer | 10 years | Yes |
| Italian  Laura | 20s | F | Stratford | Beauty therapist | 10 years | Yes |
| Iranian  Maeda | 30s | F | West London | MP secretary | 20 years | Yes |
| Iranian  Fadi | 60s | M | West London | Trader | 17 years | Yes |
| Iranian  Abdi | 30s | M | North West London | Self employed | 16 years | Yes |
| Iranian  Afsana | 30s | F | Central London | Senior data scientist | 12 years | Yes |
| Iranian  Aahil | Unwilling to disclose | | | | | |
| Iranian  Adar | 20s | M | North West London | Car salesman | 14 years | No |
| Iranian  Ahmad | 30s | M | North London | Manager | 9 years | Yes |
| Iranian  Adiba | 30s | F | West London | Student | 2 years | No |
| Iranian  Fahima | 40s | F | North London | Admin. manager | 14 years | No |
| Iranian  Arezou | 30s | F | Kent | Solicitor | 20 years | Yes |
| Iranian  Mahin | 30s | M | South London | IT manager | 10 years | Yes |
| Iranian  Safa | 30s | M | West London | Data scientist manager | 12 years | Yes |
| Iranian  Mehria | 30s | F | East London | Production accountant | 10 years | No |
| Iranian  Samar | 20s | F | West London | PhD tudent | 3 years | Yes |
| Iranian  Samira | 30s | M | North London | Project manager | 17 years | Yes |
| Iranian  Sadik | 40s | M | North East | Insurance broker | 7 years | Yes |
| Iranian  Malek | 30s | F | East London | Accountant | 10 years | No |
| Iranian  Monir | 30s | F | West London | PhD researcher | 1 years | No |
| Iranian  Zabi | 30s | F | North West London | Account manager | 17 years | Yes |
| Iranian  Uzma | 30s | F | Central London | Project manager | 3 years | No |

*All participants’ names are changed to pseudonyms.
